# A New Regularization for Deep Learning-Based Segmentation of Images with Fine Structures and Low Contrast

**DOI:** 10.3390/s23041887

**Published:** 2023-02-08

**Authors:** Jiasen Zhang, Weihong Guo

**Affiliations:** Department of Mathematics, Applied Mathematics and Statistics, Case Western Reserve University, Cleveland, OH 44106, USA

**Keywords:** image segmentation, softmax, spatial regularization, fine structure, connectivity

## Abstract

Deep learning methods have achieved outstanding results in many image processing and computer vision tasks, such as image segmentation. However, they usually do not consider spatial dependencies among pixels/voxels in the image. To obtain better results, some methods have been proposed to apply classic spatial regularization, such as total variation, into deep learning models. However, for some challenging images, especially those with fine structures and low contrast, classical regularizations are not suitable. We derived a new regularization to improve the connectivity of segmentation results and make it applicable to deep learning. Our experimental results show that for both deep learning methods and unsupervised methods, the proposed method can improve performance by increasing connectivity and dealing with low contrast and, therefore, enhance segmentation results.

## 1. Introduction

Image segmentation is one of the most fundamental and important tasks in image processing and computer vision. Image segmentation aims to divide a digital image domain into several disjoint regions so that each region is homogeneous with respect to certain characteristics. For binary case it also refers to outlining the boundaries of objects of interest so that the target objects are easier to recognize. It has been applied and studied in many areas, such as biomedical imaging and geosensing.

Different methods of image segmentation have been developed. Variational methods are one of the popular ones that usually rely on optimizing some well-designed energy functional. Many classic variational models have been proposed. For example, Mumford–Shah model [1] does simultaneous image smoothing and segmentation and represents the smoothed image as a piecewise-smooth function. Potts model [2] and Chan-Vese model [3,4] adopt piecewise constant approximation. Level set and Heaviside functions are used in Chan-Vese model to represent different regions. Heaviside function is however not convex and causes optimization to become stuck in local minima. Some methods [5] apply convexity relaxation to guarantee a global solution of the models. The models usually seek optimal segmentation by minimizing some specific energy functional. The functionals usually contain a data fitting term that penalizes the error between the approximation and original image, and one or several regularization terms which represent the mathematical assumption or expectation of the underlying solutions [6,7,8].

With the development of a fully convolutional network (FCN) and improvement of computing power, deep learning-based methods have achieved remarkable performance on image segmentation. With the images as input, the trained neural network can classify each pixel of the input images by predicting the probabilities of each class. Recently, many new deep learning models and structures have been proposed. Examples are GoogleNet [9], UNet [10], Res-Net [11], Xception [12], and DeepLab models [13,14,15,16]. There are also some combinations, such as Res-UNet [17]. Compared with variational methods, deep learning methods can extract high-level features of images, as well as low-level features such as edges by feeding a set of training images into the neural network. That allows it to extract deeper meanings of pixels and deal with more complicated tasks, such as semantic image segmentation.

On the other hand, variational methods are still popular in many areas, such as medical image processing [6], due to some advantages. First, most variational methods [1,3,4,5,18] are unsupervised or semi-supervised so that they are still available with little or without labeled data. Second, it is flexible to add many kinds of spatial regularization into the objective function [6,8], which helps variational methods obtain more reasonable results. Therefore, it is a good idea to apply spatial regularization into neural networks to combine the advantages of the two methods.

In fact, some methods adding regularizations into a neural network have been proposed. Some of them modify the loss functions by using objective functions of variational models [19] or directly adding spatial regularization [20,21] to the loss functions. Some methods make use of feature extraction and attention mechanism [22] to fuse region features and boundary features. In addition, some methods apply spatial regularization into activation functions by representing activation functions as the solutions of some variational problems [23]. There, the authors have discussed the advantages of these methods over other methods. First, by modifying the activation function, they can affect the process of back-propagation and, therefore, the learnable parameters, while the loss functions usually only affect the training stage. Second, they are flexible to be applied in any number of activation functions in any layers. In this paper, we propose a new regularity that could be used alone in non-supervised methods or adopted into activation functions. It is only used in the last layer for a better efficiency without losing much performance.

Total variation (TV), one of the most widely used spatial regularization during image segmentation, can minimize the boundary length and has the effect of removing noise. However, it is not suitable for images with low contrast, fine and multi-scale structures. Examples are blood vessels in medical images, branches of trees and antennae of insects. These images vary greatly in scale and shape. Low contrast makes it even worse as fine structures are often mistakenly treated as background and cause missing edges of fine structures. In these cases, a spatial regularization that could lengthen the boundary or enhance connectivity would help to catch boundaries of fine structures. Although some regularization methods have been proposed to improve connectivity by computing discrete curvatures for each piece of edges [24,25], they are used in variational models and are very difficult to be applied to deep learning. Moreover, it is inefficient to compute curvatures for each image and each training step in deep learning. In this paper, based on a soft thresholding dynamic (STD) regularization which can make boundary smooth if added to activation function [23], we design a new connectivity boosting regularization that is very easy to be adopted in deep learning framework.

## 2. Related Works

### 2.1. Deep Learning Based Image Segmentation

Neural networks for image segmentation consist of a series of linear and non-linear operations. After being fed into a neural network, the image passes through the hidden layers in sequence. In a simple FCN, the input or feature map of each layer is the output of the previous layer. Suppose the output of the *i*th layer is $o^{i}$ ($o^{1}$ is the input image), then the output of the next layer would be:

$$\begin{array}{c} o^{i+1}=A^{i}\left(W^{i}o^{i}+b^{i}\right)\;i=1,2,3\cdots \end{array}$$

$W^{i}$ and $b^{i}$ represent linear operation and contain learnable parameters of the *i*th layer. $A^{i}$, called activation function, is a non-linear function and usually remains unchanged. Activation functions add non-linearity to the neural network and enable the neural network to fit any non-linear functions. For image segmentation, the final output of the last layer has the same shape as the input image so that the neural network can predict the probability of each pixel belonging to each class.

In the process of training, the discrepancy between the output of training images and ground truth are computed according to a certain loss function. By applying backpropagation, the gradients of the loss function with respect to each parameter are obtained so that the neural network can ’know’ how to update the parameters. Specifically, the parameters are updated with gradient descent-based algorithms to reduce the loss.

### 2.2. Spatial Regularization in Variational Methods

As mentioned, variational methods involve minimizing some energy functions that can be simply formulated as the sum of a fidelity term and a regularization term:

(1) $$\begin{array}{cc} \; & \min_{u}F\left(u,o\right)+R\left(u\right) \end{array}$$

The fidelity term F(u,o) measures the similarity between the original image o and the segmentation result u. For example, the Euclidean distance $||u-{o||}_{2}^{2}$ is used in many variational models. The regularization term R(u) is designed to have some certain influence on the result. Many kinds of regularity terms, such as total variation, Tikhonov, total generalized variation [26] and total fractional variation [27] have been proposed. In particular, TV regularization can be expressed as the sum of the norm of gradient: |∇u|. In an image, non-zero gradient usually means appearance of boundary. Therefore, TV regularization can approximate the boundary length and the segmentation result would be more robust to noise and preserve edges better.

Although TV regularization performs well in segmentation tasks, its non-smoothness property makes it not efficient in deep learning. Typically, the variational problems with TV regularization can only be solved with some methods that are too complicated for deep learning, such as alternating direction method of multipliers (ADMM) and primal dual methods. Compared with gradient-based methods, such as gradient descent, these methods usually converge much slower and each of their steps is computationally expensive. The computation of total variation itself is time consuming too. Since the training process of deep learning can take hundreds of epochs, the regularization used in deep learning should be able to be solved efficiently.

### 2.3. Soft Threshold Dynamic (STD) Regularization

To combine deep learning framework and spatial regularizations, Liu et al. [23,28,29] have given variational explanations for some widely used activation functions in deep learning including softmax, ReLU, and sigmoid [23,28,29]. For example, softmax operator can be written as $\mathrm{softmax}{\left(o\right)}_{i}=\frac{\exp(o_{i})}{\sum_{i=1}^{I}\exp\left(o_{i}\right)}$ and it can be represented as the solution of the following optimization problem [23] when the parameter ε is 1:

(2) $$\begin{array}{cc} \; & \min_{u\in U}\langle -o,u\rangle +\varepsilon \langle u,\ln u\rangle \;U=\left\{u\in {\left[0,1\right]}^{C}:\sum_{i=1}^{C}u_{i}\left(x\right)=1,\forall x\in \Omega \right\} \end{array}$$

where Ω is the domain of the whole image, *C* is the number of classes, o is the feature map input, and u is the output of the softmax operator. Although problem (2) has no regularization about u, one can easily add regularization terms used in image processing and computer vision. In [23], soft threshold dynamics (STD) [30] was adopted:

(3) $$u^{*}=\underset{u\in U}{\arg\;\min}\langle -o,u\rangle +\varepsilon \langle u,\ln u\rangle +\lambda \langle u,k*\left(1-u\right)\rangle$$

where ∗ means convolution, *k* represents a discrete Gaussian kernel and λ is a parameter. The STD regularization term 〈u,k∗(1−u)〉 comes from convolution generated motion [31,32] whose goal is to simulate the motion of interfaces of some dynamic systems, such as chemical and biological systems. When simulating the dynamics of interface under surface tensions [30], it assumes that the interfacial energy is proportional to the length of interface. Thus, STD term estimates the boundary length.

Compared with TV regularization, STD regularization has similar effect. However, it is smooth and, thus, problem (3) can be solved with much more efficient algorithms. Furthermore, STD regularization involves convolution and inner product operation, which are cheaper to calculate than norm.

Theoretically, STD regularization can be added to arbitrary numbers of activation functions in any layers in a neural network, but in this paper, the proposed regularization to the softmax function is just applied in the last layer for some reasons. First, solving the optimization problems for many layers is time consuming because most of them can only be solved with iterative methods. Second, the numerous feature maps in the hidden layers represent certain features of the image. Applying regularization in these layers may have unknown influence on the extraction of the features. In practice, the parameters ε, λ, and σ can be set as learnable so that they can be tuned automatically by neural network.

## 3. Proposed Method

### 3.1. Explanation of STD Regularization

**Figure 1:**
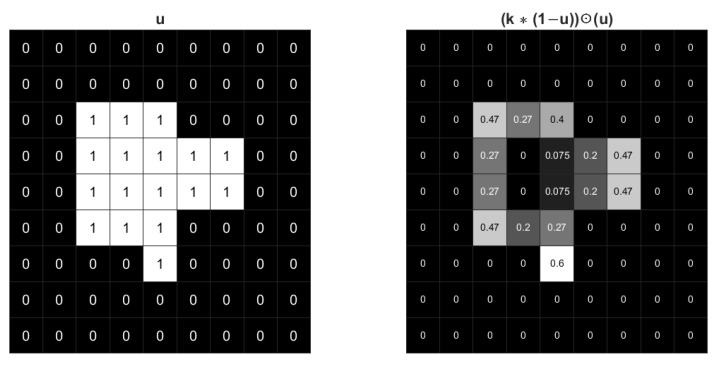
The effect of a 3×3 Gaussian kernel with σ=1 for an image. The gray value shades correspond to the values of each pixel.

The STD regularization term 〈u,k∗(1−u)〉 is an approximation of boundary length while used in image segmentation model. By computing the convolution of the image with a Gaussian kernel, a series of weights related to curvature can be generated along the boundaries of different regions. The sum of these weights along the boundary can be a good approximation of the boundary length. To help understand STD regularization and illustrate the effect of Gaussian kernel, an example of binary u with values 0 and 1 is shown in Figure 1. Only pixels along the boundary of u have non-zero values in u⊙k∗(1−u). Additionally, the inner product 〈u,k∗(1−u)〉 is the summation of u⊙k∗(1−u). The pixels along the corners have higher values, which means 〈u,k∗(1−u)〉 has larger weight when the curvature is higher. Therefore, the regularization is an approximation of boundary length weighted by curvature.

Note that the Gaussian kernel in STD makes the regularization penalize arc length in all directions. In certain instances, however, we need to enhance connectivity which leads to longer arc length along certain directions. For instance, when the dominant edges of an image are horizontal, we should allow long arc length along the horizontal direction (see Section 4.2 for one example). Therefore, it is necessary to design a new regularization that can enhance the connectivity in a selective way.

### 3.2. New Regularization Term

To enhance connectivity, a new regularization term is designed to expand the foreground. Naturally, suppose the values of u on the foreground are close to one, the regularization term can be formulated as

(4) $$\begin{array}{c} \langle u,k*(-u)\rangle \end{array}$$

**Figure 2:**
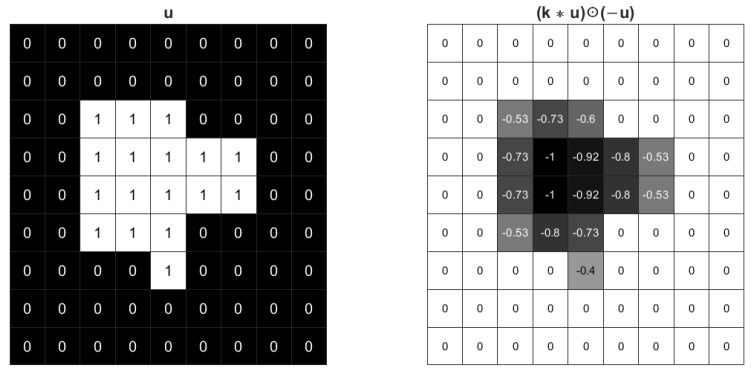
The effect of a 3×3 Gaussian kernel with σ=1 for an image.

Figure 2 shows the effect of the regularization term (4) with Gaussian kernel. The foreground value becomes −1 and the boundary values are also negative. So the inner product should be the minus sum of weighted boundary length and foreground area. The regularization (4) tends to expand the area of the foreground in a rate weighted by the boundary curvature. The foreground will not expand without limit due to the existence of loss function and tuned parameters.

**Figure 3:**
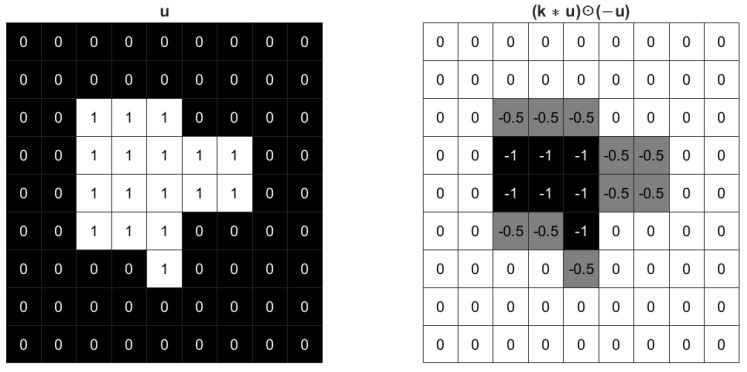
The effect of a 3×3 vertical kernel $k_{1}$ for an image.

In practice, it would be better to enhance connectivity and elongate boundary length in a certain direction, and it is not difficult to design such kernels. For example, a new 3×3 kernel can be defined as shown in (5). In Figure 3, $u⊙k_{1}*\left(-u\right)$ has larger weights on horizontal boundary, therefore when expanding the foreground it has higher priority to elongate vertical boundary.

(5) $$\begin{array}{c} k_{1}=\left[\begin{array}{ccc} 0 & 0.5 & 0\\ 0 & 0 & 0\\ 0 & 0.5 & 0 \end{array}\right] \end{array}$$

Similarly, we can design such kernels in three other directions: horizontal, 45 degree, and 135 degree diagonals.

$$k_{2}=\left[\begin{array}{ccc} 0 & 0 & 0\\ 0.5 & 0 & 0.5\\ 0 & 0 & 0 \end{array}\right]\;k_{3}=\left[\begin{array}{ccc} 0.5 & 0 & 0\\ 0 & 0 & 0\\ 0 & 0 & 0.5 \end{array}\right]\;k_{4}=\left[\begin{array}{ccc} 0 & 0 & 0.5\\ 0 & 0 & 0\\ 0.5 & 0 & 0 \end{array}\right]$$

### 3.3. Proposed Model

To better handle images with low contrast multi-scale features that could be disconnected by existing segmentation method, the new regularity is added to the model (3) to obtain the model (6). The parameters $\lambda_{i}$ and λ are balancing weights. In deep learning framework, they can be set as learnable parameters so that the weights can be adjusted continuously. Notice that we remain the STD regularization term (4th term) to address noise. One can choose to remove or retain STD for different problems. Generally, it should be retained if the noise in background is strong.

We also note that all the four kernels in the model are used. Our deep learning framework can, however, automatically update weights $\lambda_{i}$ and they will be positive and different, with the largest one corresponding to the the dominant direction of connectivity to enhance (see one example and the explanation in Section 4.2).

(6) $$u^{*}=\underset{u\in U}{\arg\;\min}\langle -o,u\rangle +\varepsilon \langle u,\ln u\rangle +\sum_{i=1}^{4}\lambda_{i}\langle u,k_{i}*\left(-u\right)\rangle +\lambda \langle u,k*\left(1-u\right)\rangle$$

The objective function is the sum of a series of smooth functions. The kernels $k_{1}$, $k_{2}$, and $k_{3}$ are semi-positive definite while $k_{4}$ is indefinite. In most cases, the weighted sum of kernels is semi-positive definite, and the regularization term is concave. Then the problem (6) can be solved with the iterative method proposed by [23]:

(7) $$u^{t+1}=\underset{u\in U}{\arg\;\min}\langle -o,u\rangle +\varepsilon \langle u,\ln u\rangle +\langle p^{t},u-u^{t}\rangle$$

where $p^{t}=\sum_{i=1}^{4}\lambda_{i}k_{i}*\left(-2u^{t}\right)+\lambda k*\left(1-2u^{t}\right)\in \partial R\left(u^{t}\right)$ and $\partial R(u^{t})$ is the subgradient of the sum of the 3rd and 4th term in the model (6). The solution of each step is:

(8) $$\begin{array}{c} u^{t+1}=\mathrm{softmax}\left(\left(o-p^{t}\right)/\varepsilon \right) \end{array}$$

When $\lambda_{4}$ is too large the weighted sum of kernels may not be strictly semi-positive definite. The general model can be solved with Proximal Forward–Backward Scheme (PFB) [33,34] efficiently. It converges quickly for a wide range of non-convex problems whose objective function is the sum of smooth and non-smooth functions without assuming convexity. The iterative algorithm with PFB in the t-th step is:

(9) $$u^{t+1}=\underset{u}{\arg\;\min}\langle -o,u\rangle +\varepsilon \langle u,\ln u\rangle +\langle p^{t},u-u^{t}\rangle +\frac{\tau }{2}||u-u^{t}{\left|\right|}^{2}$$

where τ is a constant to be tuned. The closed form solution can be represented with Lambert W function *W*:

(10) $$\begin{array}{cc} u^{t+1} & =\frac{\varepsilon }{\tau }W\left(\frac{\tau }{\varepsilon }\exp\left(\frac{o-\varepsilon I+-p^{t}+\tau u^{t}}{\varepsilon }\right)\right) \end{array}$$

where *I* is a tensor of the same size as u with all the entries equal to one. In practice, the value of Lambert W function can be well approximated by Winitzki’s approximation [35]. In the final step, to make sure the constraint is satisfied, one more softmax operation on u is performed. Both of the methods will converge within about 10 steps.

## 4. Results

For the purpose of testing whether the proposed model could enhance connectivity in image segmentation with low contrast, we focus on crack images and retinal vessel images for their difficulty in obtaining connected fine structures under the condition of low contrast. First we use the Crack Forest Dataset [36] including 118 forest images of size 448×448. In total, 18 images are randomly selected as the test set. The tested dataset of retina vessel segmentation is DRIVE [37], which is one of the most frequently used retina vessel datasets. It consists of 40 color images of size 565×584, 20 of which are used as the training set. Both two datasets vary greatly in scale and shape with low contrast. In addition, two images are used to test unsupervised image segmentation, respectively. STD regularization is used for the Crack Forest Dataset.

The deep learning model is based on U-Net whose convolution layers are replaced by depthwise separable convolution layers with residual connections used in Xception [12] architectures. Such a structure can largely improve the performance of the original U-Net. Preprocessing includes random flip, randomcrop, and contrast limited adaptive histogram equalization (CLAHE) [38]. Both random flip and randomcrop are popular data augmentation techniques by artificially expanding the size of dataset. They can artificially expand the size of dataset and avoid overfitting [39], which is especially helpful for training dataset of small size. CLAHE is a powerful method of image enhancement and has been proved to be able to improve the quality of retina vessel and crack images [40,41]. Since the preprocessing, initial parameters and shuffle of mini batch in deep learning can produce some randomness, for each dataset, we calculate the average values and standard deviations of five computations.

### 4.1. Evaluation Metrics

The results are compared and evaluated with some commonly used metrics including accuracy (Acc), precision (Pre), sensitivity (Sen), specificity (Spe), F1 score (F1), and AUC, i.e., the area under the receiving operator characteristic (ROC) curve. In binary classification they are defined as:

(11) $$\begin{array}{cc} \; & \mathrm{Acc}=\frac{\mathrm{TP}+\mathrm{TN}}{\mathrm{TP}+\mathrm{TN}+\mathrm{FP}+\mathrm{FN}} \end{array}$$

(12) $$\begin{array}{cc} \; & \mathrm{Pre}=\frac{\mathrm{TP}}{\mathrm{TP}+\mathrm{FP}} \end{array}$$

(13) $$\begin{array}{cc} \; & \mathrm{Sen}=\frac{\mathrm{TP}}{\mathrm{TP}+\mathrm{FN}} \end{array}$$

(14) $$\begin{array}{cc} \; & \mathrm{Spe}=\frac{\mathrm{TN}}{\mathrm{TN}+\mathrm{FP}} \end{array}$$

(15) $$\begin{array}{cc} \; & F1=\frac{2*\mathrm{Sen}*\mathrm{Pre}}{\mathrm{Sen}+\mathrm{Pre}} \end{array}$$

where TP is true positive, i.e., the number of truly classified positive pixels representing the foreground or the vessels. Similarly, TN, FP, and FN are true negative, false positive, and false negative. AUC is the area under the curve created by 1 − Spe in the x axis and Sen in the y axis.

Sensitivity is the accuracy rate for the foreground. Specificity is the accuracy for the background. A higher sensitivity means better connectivity for the vessels while the effect of specificity is inverse. Therefore, the main effect of my model is increasing the sensitivity at the cost of slightly decreasing specificity.

### 4.2. Results and Discussion

**Figure 4:**
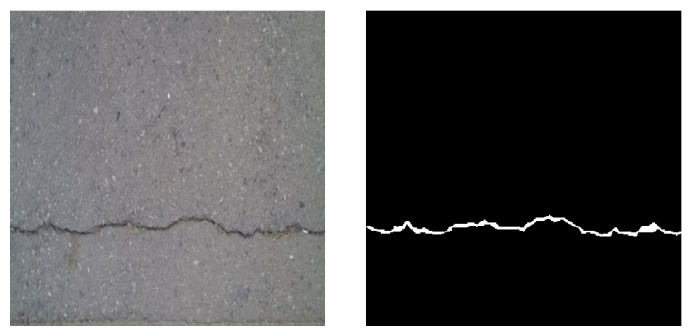
An example of the crack images. (**Left**) image. (**Right**) ground truth.

**Figure 5:**
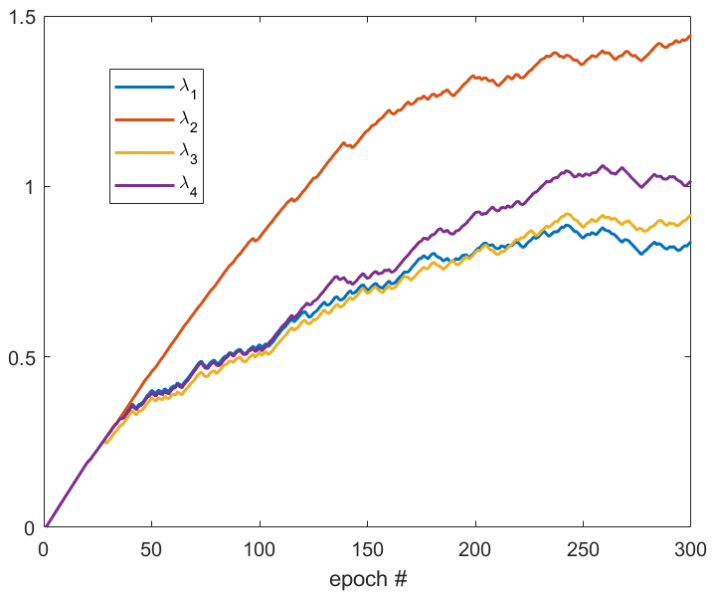
Development of weights of different kernels.

First, the development of $\lambda_{i}$’s is tested with 30 selected crack images. These images are selected from Crack Forest Dataset in which the cracks are all in horizontal direction (Figure 4). As shown in Figure 5, after 50 epochs, the value of $\lambda_{2}$ becomes the smallest so that the weight of $k_{2}$ is the smallest. Note that $k_{2}$ corresponds to the horizontal kernel, and, therefore, the trained network tends to enhance the connectivity in horizontal direction. This example shows that the neural network can automatically decide the dominant direction of the boundary.

#### 4.2.1. Crack Forest Dataset

**Figure 6:**
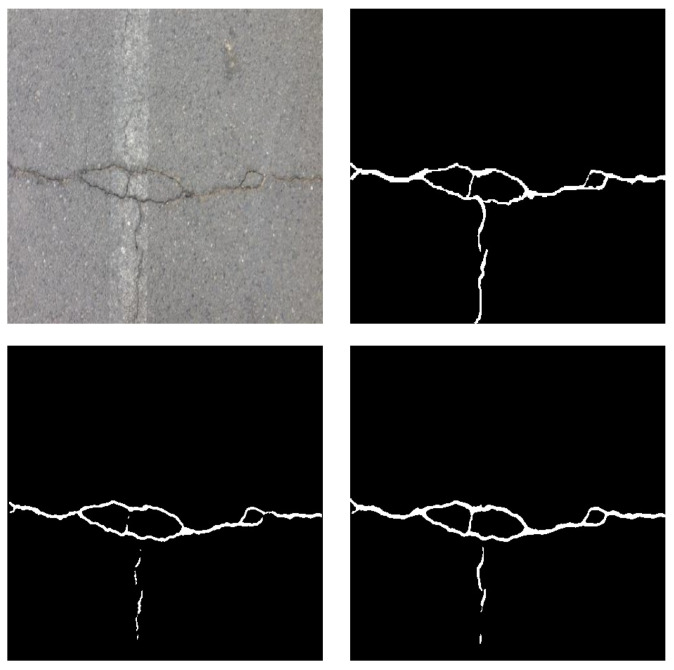
An example of the crack images. (**Top left**) image. (**Top right**) ground truth. (**Bottom left**) without regularization. (**Bottom right**) with proposed regularization.

First, the full Crack Forest Dataset is tested. The performance with or without the proposed regularization is compared in Table 1. The proposed regularization improves the performance of the first four metrics except AUC. Specifically, as we expected more pixels are classified as foreground, therefore the regularization can obviously increase sensitivity and thus improve connectivity. An example shown in Figure 6 more explicitly illustrates the improvement. In the bottom right, while preserving the main part of the crack like the bottom left, the proposed regularization helps recognize more details of small branches and connect some disconnected parts.

#### 4.2.2. Retina Vessel

**Figure 7:**
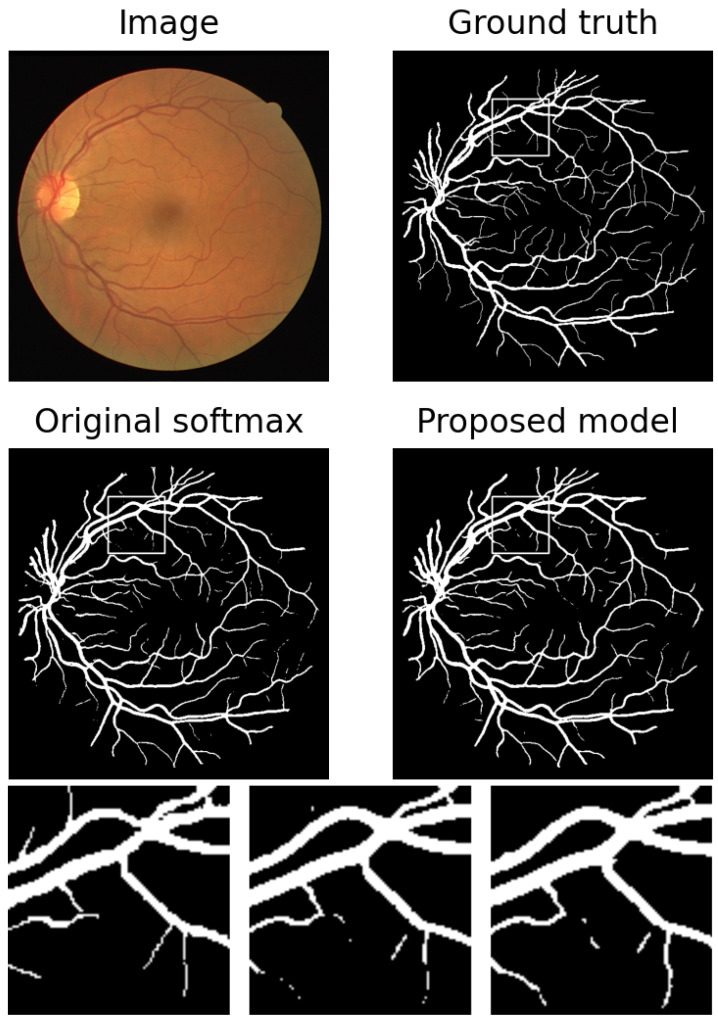
An example of results in Table 2. From left to right: ground truth, results with original softmax, and results with proposed regularization.

We test the performance of our model on DRIVE dataset. The comparison of results with and without the proposed regularization are shown in Table 2. As expected, the sensitivity is increased at a cost of slightly reduced specificity. While the accuracy and F1 score almost remain unchanged, the AUC is largely improved. An example is shown in Figure 7, and a zoomed region indicated by white boxes is shown too. Some missing branches are elongated, and some disconnected parts are connected. In Table 3, our results are compared with some state-of-the-art methods. Our accuracy and specificity are the highest among them and our sensitivity and AUC are also comparable to them. F1 score is not included because few of the papers show it. Even better performance are expected if we apply attention mechanism or more pre- and post-processing techniques.

#### 4.2.3. Unsupervised Model

**Figure 8:**
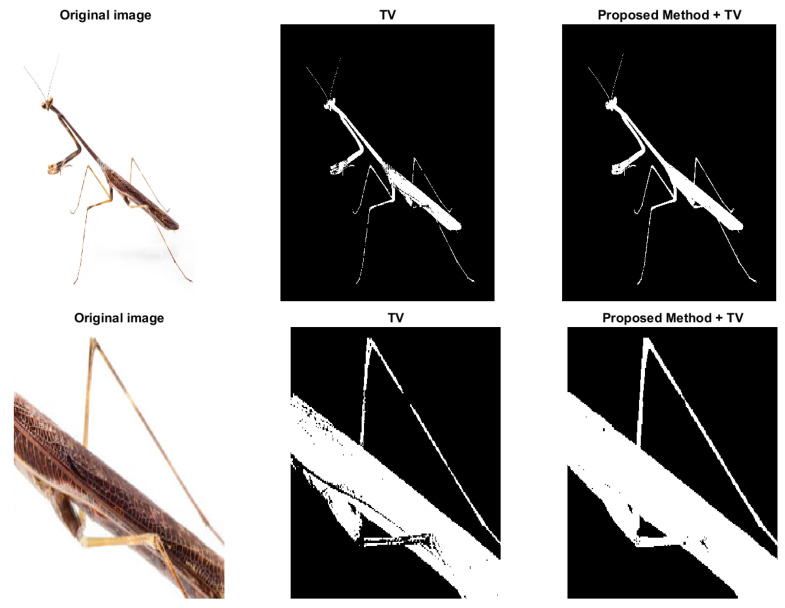
The effect of the proposed regularization on unsupervised model. Column 2: the model in [5] with TV regularization only. Column 3: the proposed model (16).

**Figure 9:**
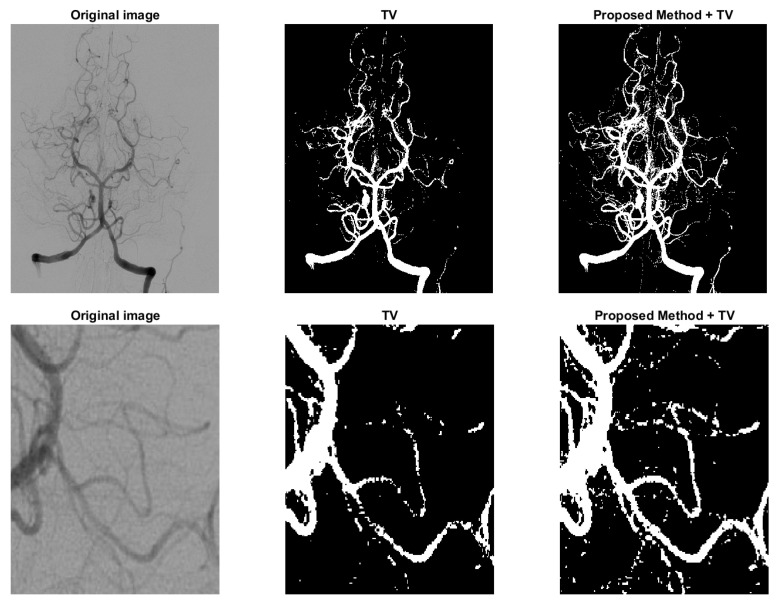
Another example to show the effect of the proposed regularization on unsupervised model. Column 2: the model in [5] with TV regularization only. Column 3: the proposed model (16).

To show the effectiveness of the proposed regularization term, we add it to the energy function in the variational model [5] that only involves total variation. The optimization problem can be formulated as:

(16) $$\underset{u\in U}{\arg\;\min}\langle u,f_{1}-f_{2}\rangle +\lambda_{1}\int_{\Omega }\left|\nabla u\right|dx+\lambda_{2}\langle u,k*\left(-u\right)\rangle$$

where $f_{1}={\left(f-c_{1}\right)}^{2}$ and $f_{2}={\left(f-c_{2}\right)}^{2}$, *f* represents the image and $c_{1}$ and $c_{2}$ are the means of the foreground and background indicated by u. Because in unsupervised method the parameter λ’s are fixed, we just apply Gaussian kernel in the regularization added with TV. Since the added regularization term is smooth, the modified model can be solved with commonly used methods, such as ADMM. The proposed model (16) is compared with the one in [5] and some results are shown in Figure 8 and Figure 9. Figure 8 is a typical example of images with multi-scale features, the legs of the insect are long and slim, and some parts of the insect mix with the background due to low contrast. In variational models these parts with low contrast are naturally recognized as background. However, in the third figure of Figure 8, the legs are connected as a whole and some noise on the body caused by the texture pattern are removed. Another example is shown in Figure 9, which is human arteries. The image noise is strong and the contrast between the vessels and the background is very low. As shown in the second column of Figure 9, if the effect of TV is strong, the model will eliminate some fine structures together with noise. However, in the third column, the model retains some vessels, and we can find the corresponding parts from the original image in the first column. The parameters $\lambda_{1}$ and $\lambda_{2}$ in the Equation (16) should be fine-tuned. Typically, $\lambda_{1}$ is between 0.01 to 1. $\lambda_{2}$ should be as small as $10^{-6}$. Both results show the potential of the proposed regularization in enhancing segmentation of images with multi-scale structures and low contrast.

## 5. Conclusions

A novel spatial regularization for image segmentation is proposed, which can be applied flexibly in neural networks. It is designed to enhance segmentation of fine structures especially in images with low contrasts through improving connectivity. During process of training the neural network can learn to find the dominant direction of the boundary. Our results show that the proposed regularization can improve the performance of neural network on some suitable datasets. Specifically, we test the retina vessel and forest crack datasets and achieve better results compared with some recently proposed models. We observe obvious improvements of sensitivity corresponding to better connectivity. In addition, the effect of the proposed regularization applied in an unsupervised model is tested and we find improvement. In the future, we will focus on improving connectivity locally. Additionally, we will continue to design other specific spatial regularizations based on similar mechanism.

## Figures and Tables

**Table 1:** Results of CFD dataset with and without regularization.

Metrics	Without Regularization	Proposed Regularization
Acc	0.9911 ± 0.0002	0.9914 ± 0.0001
Pre	0.6997 ± 0.0229	0.7131 ± 0.0256
Sen	0.6489 ± 0.0359	0.6582 ± 0.0567
Spe	0.9960 ± 0.0007	0.9962 ± 0.0008
F1	0.6627 ± 0.0093	0.6747 ± 0.0210
AUC	0.9600 ± 0.0117	0.9548 ± 0.0131

**Table 2:** Results of DRIVE with and without regularization.

Metrics	Without Regularization	Proposed Regularization
Acc	0.9685 ± 0.0002	0.9680 ± 0.0002
Pre	0.843 ± 0.0108	0.8192 ± 0.0038
Sen	0.7930 ± 0.0128	0.8205 ± 0.0048
Spe	0.9857 ± 0.0014	0.9824 ± 0.0005
F1	0.8140 ± 0.0019	0.8167 ± 0.0012
AUC	0.9484 ± 0.0042	0.9760 ± 0.0029

**Table 3:** Performance of different models on DRIVE. The best values are boldened.

Method	Acc	Sen	Spe	AUC
U-Net [10]	0.9656	0.8132	0.9805	0.9430
DeepLabV3+ [16]	0.9391	0.6950	0.9628	0.9213
R2U-Net [42]	0.9556	0.7792	0.9813	0.9784
Vessel-Net [43]	0.9578	0.8038	0.9802	**0.9821**
DUNet [44]	0.9566	0.7963	0.9800	0.9802
CE-Net [45]	0.9545	0.8309	0.9747	0.9779
Pyramid U-Net [46]	0.9615	0.8213	0.9807	0.9815
DCU-Net [47]	0.9568	0.8115	0.9780	0.981
CSAU-Net [48]	0.9676	**0.834**	0.981	0.9758
Our results	**0.9680**	0.8205	**0.9824**	0.9760

## Data Availability

Not applicable.
